# Analysis of an Attractor Neural Network’s Response to Conflicting External Inputs

**DOI:** 10.1186/s13408-018-0061-0

**Published:** 2018-05-16

**Authors:** Kathryn Hedrick, Kechen Zhang

**Affiliations:** 1 Southern Methodist University, Dallas, USA; 2 Johns Hopkins University, Baltimore, USA

## Abstract

The theory of attractor neural networks has been influential in our understanding of the neural processes underlying spatial, declarative, and episodic memory. Many theoretical studies focus on the inherent properties of an attractor, such as its structure and capacity. Relatively little is known about how an attractor neural network responds to external inputs, which often carry conflicting information about a stimulus. In this paper we analyze the behavior of an attractor neural network driven by two conflicting external inputs. Our focus is on analyzing the emergent properties of the megamap model, a quasi-continuous attractor network in which place cells are flexibly recombined to represent a large spatial environment. In this model, the system shows a sharp transition from the winner-take-all mode, which is characteristic of standard continuous attractor neural networks, to a combinatorial mode in which the equilibrium activity pattern combines embedded attractor states in response to conflicting external inputs. We derive a numerical test for determining the operational mode of the system *a priori*. We then derive a linear transformation from the full megamap model with thousands of neurons to a reduced 2-unit model that has similar qualitative behavior. Our analysis of the reduced model and explicit expressions relating the parameters of the reduced model to the megamap elucidate the conditions under which the combinatorial mode emerges and the dynamics in each mode given the relative strength of the attractor network and the relative strength of the two conflicting inputs. Although we focus on a particular attractor network model, we describe a set of conditions under which our analysis can be applied to more general attractor neural networks.

## Introduction

The theory of attractor neural networks has greatly influenced our understanding of the mechanisms underlying the computations performed by neural networks. This is especially true for hippocampal networks involved in spatial, declarative, and episodic memory. According to this theory, structured recurrent connections among *N* neurons cause the *N*-dimensional state vector to converge in time to a stable, low-dimensional space called the attractor [1]. Such a network embeds memories as stationary attractors, which may be a discrete set of point attractors representing a discrete set of objects [2] or a continuum of attractor states representing continuous variables such as heading direction [3, 4] or spatial location within an environment [5–10]. Numerous theoretical studies have revealed properties of attractor neural networks that make them a desirable neural mechanism for memory storage, such as robustness to damage, pattern completion, and generalization [11, 12]. Attractor neural networks should arise naturally in regions of the brain with recurrently connected neurons and Hebbian-type synaptic plasticity, and they provide a theoretical framework for experimental design and data interpretation [13].

Attractor neural networks have been studied extensively through both analysis and computational simulations [1, 3, 14–17]. While some studies do examine the role of external input [16, 18, 19], most determine the set of stable equilibrium states in the absence of external input, establishing properties such as the structure and capacity of the attractor. Relatively little is known about how an attractor network may respond to conflicting external inputs. This creates a gap between the idealistic predictions of attractor network theory and experimental data, since it is often experimentally difficult if not impossible to isolate putative attractor dynamics from the influence of the strong (often conflicting) external inputs into the neural network. In the current study, we analyze an attractor neural network’s response to conflicting external inputs that effectively create a competition between embedded attractor states. Our focus is the interesting behavior observed in our numerical simulations of the megamap model, a quasi-continuous attractor network representing a large spatial environment, driven by external inputs encoding two different locations in the environment [10]. However, the analytical methods and results obtained here can be applied to more general attractor network models.

The megamap model is designed for a network of principal cells in the CA3 subregion of the hippocampus, a region crucial for learning and memory [20–22]. These cells are often referred to as place cells due the strong spatial correlate of their activity. In small, standard recording environments (∼1 m^2^), a given place cell is primarily active when the animal is within one specific subregion of the environment, called the cell’s place field [21, 23]. The megamap model flexibly recombines place cells to extend standard attractor network models of place cells, in which the majority of cells have one place field, to larger environments in which place cells have been shown experimentally to have multiple, irregularly spaced place fields [24–26]. The model follows logically from the recurrent connections among place cells in the CA3 [27], the Hebbian-like associative plasticity observed in the hippocampus [12, 28, 29], and the consistent co-activity of place cells with neighboring place fields [30].

Since the megamap seamlessly represents much larger environments than is possible for standard attractor network models of place cells, it allows us to explore whether any interesting dynamics emerge in large environments. In our numerical simulations, we observed a sharp transition in the network’s response to conflicting external inputs as the environment continuously grew in size [10]. In relatively small environments, the megamap behaves similarly to standard continuous attractor neural networks, operating in the *winner-take-all (WTA) mode* whereby the equilibrium state fully represents one input while effectively ignoring the second input. In larger environments, the megamap operates in the *combinatorial mode*, effectively combining two embedded attractor states to stably represent both inputs. Furthermore, we observed hysteresis, a classic characteristic of attractor dynamics, in the WTA mode, but the initial state had no effect on the equilibrium state in the combinatorial mode. The combinatorial mode is an interesting emergent property of the model that may be related to the partial remapping of hippocampal place cells sometimes observed when an animal is introduced to a new environment that simultaneously resembles two different familiar environments. In this cue conflict situation, the evoked neural responses are often mixtures of the responses to both environments rather than representations of one environment only [31]. The combinatorial mode emerges in the megamap model in sufficiently large environments when the weights are set optimally through gradient descent but not when the weights are set by the basic Hebbian learning rule [32, 33]. The latter method is widely used in attractor network models of place cells representing multiple environments [5, 6, 34–36].

We previously explored this emergent property of the megamap through numerical simulations and discussed its implications [10]. In the current study, we use mathematical analysis to derive a numerical test for determining the operational mode of the system *a priori*, characterize the conditions under which the combinatorial mode emerges, and derive explicit equations for the parameters of the model at which bifurcations occur. The numerical test is derived through stability analysis. It is an easily applied, useful tool for determining the expected response of a general attractor network to conflicting external inputs. This is particularly useful when the attractor network is self-organized. The latter two results are obtained through a linear mapping of the *N*-dimensional dynamical system to a 2-dimension reduced model. Analysis of the stable fixed points of the reduced model elucidates the attractor network strength, which we quantify, and the relative strength of conflicting external inputs for which the equilibrium state vector represents the first location, represents the second location, represents one location or the other dependent on the initial state (hysteresis), or represents both locations. The explicit equations relating the dynamics of the attractor network to the model parameters are particularly useful when designing an attractor network to model a set of observed phenomena.

An outline of the paper is as follows. In Sect. 2, we present the dynamical system of the megamap model and describe two methods used to set the recurrent weights. We then show a numerical example of the operational modes and derive a numerical test for determining the operational mode. In Sect. 3, we present the reduced 2-unit model and describe the conditions under which the reduced model is an accurate approximation of the full attractor network model. In Sect. 4, we characterize the conditions under which the combinatorial mode emerges and derive equations for the bifurcations of the dynamical system. We close in Sect. 5 by comparing our analysis to other analytical treatments of attractor neural networks, describing possible extensions of the reduced model, and discussing the implications of the results for various types of attractor network models.

## Operational Modes of the Megamap

We begin by describing the basic equations governing the megamap model and by illustrating the operational modes through a numerical example. For further details, see [10].

### Megamap Model

The megamap model is a standard firing rate model [18] consisting of a network of *N* place cells with recurrent excitation, global feedback inhibition, and external input. The state vector, $\textbf{u}\in \mathbb{R}^{N}$, loosely represents the depolarization of each place cell and is governed by

1 $$\tau {\text{u}}^{\prime }(t)=-\text{u}(t)+\text{W}f\left(\text{u}(t)\right)-w^{I}f^{I}\left(\text{u}(t)\right)1+\text{b},$$

where $\tau=10\mbox{ ms}$ for all simulations, and $1\in R^{N}$ denotes a vector of all ones. Our interest is in how the activity vector, $f(\textbf{u})\in \mathbb{R}^{N}$, is tuned to spatial location. For simplicity, we set the activity through the threshold linear gain function, $f(\textbf{u}) = f_{\mathrm{pk}} [[u_{1}]_{+},\ldots,[u_{N}]_{+}] ^{\mathsf{T}}$, where $[\cdot]_{+} = \max(\cdot,0)$, and $f_{\mathrm{pk}}= 15\mbox{ Hz}$ is the peak firing rate of the activity bump. All interneurons are modeled as a single inhibitory unit providing global feedback inhibition so that only the external input and recurrent hippocampal input provide a spatial signal. The activity of the inhibitory unit is given by $f^{I}(\text{u})={\left[1^{T}f(\text{u})-\theta \bar{f_{\mathrm{net}}}\right]}_{+}$, where *θ* is the threshold parameter, and $\overline {f_{\mathrm {net}}}= \sum_{i=1}^{N} \overline {f}_{i}(\textbf{x})$ is the sum over any embedded activity pattern (Eq. (2)). The embedded activity patterns are set such that $\overline {f_{\mathrm{net}}}$ is independent of **x**. The inhibitory activity is scaled by the inhibitory weight parameter, $w^{\mathrm{I}}$. The external input, $\textbf {b}\in \mathbb{R}^{N}$, carries sensory information about the animal’s location or self-motion, modeling idealistic neuronal inputs from the upstream entorhinal cortex.

The recurrent excitation, $\textbf{W}f(\textbf{u})$, provides the internal network drive. The weight matrix, $\textbf{W}\in \mathbb{R}^{N\times N}$, represents the strength of connections among place cells. Several studies have shown that an attractor network emerges in relatively small environments (∼1 m^2^) when the weights are set through Hebbian plasticity [9, 34]. We constructed a benchmark model for how an attractor network of place cells can represent large spaces by setting the weights to obtain desired activity profiles (place fields) for each cell [10]. The preferred locations (place field centers) for each cell are distributed randomly throughout the environment, and the number of place fields per cell is set according to the Poisson distribution. The average density of place fields for a given cell is set such that 80% of place cells are silent in a 1 m^2^ environment. The weight matrix is then set in one of two ways:

1. The optimal weights are set incrementally through the delta rule [33] so that a set of desired activity patterns, $\{\overline {\textbf{f}}(\textbf{x}_{j})\}$, are embedded into the network as stable fixed points of the dynamical system (Eq. (1)) when the external input into each cell is an idealistic sum of Gaussians centered at the preferred locations of each cell (Fig. 1(a)). The desired activity of each cell is the sum of Gaussian-like place fields. Explicitly, for each cell *i* with $M_{i}$ place fields centered at $\{\textbf{c}_{im}\}_{m=1}^{M_{i}}$, the training input and desired activity are, respectively, given by
  2 $$ \begin{aligned} \overline {b}_{i}(\textbf{x}) &= \overline {b_{\mathrm{pk}}} \sum_{m=1}^{M_{i}} \exp\biggl(\frac{-|\textbf{x}-\textbf{c}_{im}|^{2}}{2\sigma^{2}} \biggr)\quad\mathrm{and}\\ \overline {f}_{i}(\textbf{x}) &= \sum_{m=1}^{M_{i}} f \biggl( (1+u_{0})\exp\biggl(\frac{-|\textbf{x}-\textbf {c}_{im}|^{2}}{2\sigma^{2}} \biggr) - u_{0} \biggr) \end{aligned} $$
  when the animal is stationary at location **x**. The training input is set as the idealistic sum of Gaussian bumps whose amplitudes are given by the parameter $\overline {b_{\mathrm{pk}}}$. The desired activity is set as the sum of activity bumps of height $f_{\mathrm{pk}}$ over each place field center. The shift parameter, $u_{0}>0$, is the depolarization at which a cell becomes active. The optimal weights are set using a discrete set of locations $\{\textbf{x}_{j}\}$ distributed uniformly over the environment (at least 15 cm from a boundary).

**Fig. 1:**
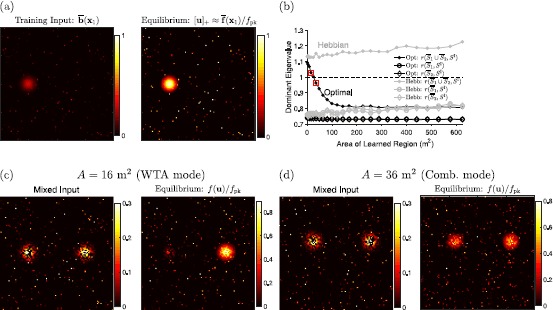
Activity bumps on the megamap. (**a**) When driven by the training input, $\overline {\textbf{b}}(\textbf{x}_{1})$, the equilibrium state corresponds to a localized activity bump well approximated by the embedded activity pattern, $\overline {\textbf{f}}(\textbf{x}_{1})$ (Eq. (2)). The training input and activity bump are visualized by plotting $b_{i}$ and $f(u_{i})/f_{\mathrm{pk}}$ for each place cell *i* redundantly at each of its preferred locations (Fig. 2(a)–(b)). (**b**) The numerical test for the operational mode (Eq. (3)) predicts that the optimal megamap transitions from the WTA mode to the combinatorial mode at about 25 m^2^, while the Hebbian megamap is always in the WTA mode. The filled circles indicate the values of $r( \overline {S_{1}}\cup \overline {S_{2}},S^{\mathrm{I}})$ (Eq. (3)) for the optimal (black) and Hebbian (gray) weights, where $\overline {S_{k}}$ is the set of all cells active in the embedded pattern at location $\textbf{x}_{k}$ $(\overline {\textbf{f}}(\textbf{x}_{k}))$, and $S^{ \mathrm{I}}=\{\mathrm{inh}\}$ since the inhibitory unit is active. The two squared points indicate values for the megamaps simulated in (**c**) and (**d**). The open circles and diamonds indicate the values of $r( \overline {S}_{k},\{\mathrm{inh}\})$, or Eq. (3) evaluated at any activity bump proportional to exactly one embedded activity pattern. All such activity bumps are stable. The “Dominant Eigenvalue” refers to the maximal eigenvalue computed in Eq. (3). (**c**) When the optimal megamap representing 16 m^2^ is driven by a mixed external input (left), only one prominent activity bump persists in time (right). The external input is formed by choosing two well-separated locations $\textbf{x}_{1}$ and $\textbf{x}_{2}$, setting $b_{i} = \overline {b}_{i}( \textbf{x}_{1})$ for a randomly selected 50% of the cells, and setting $b_{i} = \overline {b}_{i}(\textbf{x}_{2})$ for the remaining cells. The activity bump scaled by $(1/f_{\mathrm{pk}})$ is equivalent to $[\textbf{u}]_{+}$. (**d**) When the optimal megamap representing 36 m^2^ is driven by an external input set in the same manner, activity bumps representing both locations persist in time
2. The Hebbian weights are set as the sum of tuning curves,
  $$W_{jk} = W_{kj} = \sum_{m=1}^{M_{j}} \sum_{n=1}^{M_{k}} w_{\mathrm{tune}}\bigl(| \textbf{c}_{jm}-\textbf{c}_{kn}|\bigr), $$
  where each cell *j* has the preferred locations $\{\textbf{c}_{jm}\} _{m=1}^{M_{j}}$, and $w_{\mathrm{tune}}$ is the weight profile determined by computing the optimal weights when each cell has exactly one place field. This tuning curve is approximately Gaussian, and setting weights as the sum of Gaussians is a common method for constructing attractor network models of place cells [5, 6, 34–36]. The resulting weights approximate the weights expected given the basic Hebbian learning rule [32–34].

If each cell had at most one place field, then the two methods would be equivalent. Both methods lead to an attractor network that robustly represents large spaces (∼100 m^2^). Differences emerge in large environments (>16 m^2^) in which individual place cells represent multiple, irregularly spaced locations.

### Numerical Example of the Operational Modes of the Megamap

Since the megamap can seamlessly represent much larger environments than was previously possible, the model allows one to explore whether any interesting properties emerge when the attractor network represents a large space. We found that the megamap with optimal weights sharply transitions from a winner-take-all (WTA) mode to a combinatorial mode as the environment becomes sufficiently large [10]. While a megamap in either mode is similarly robust to a noisy or incomplete external input, there are clear differences between the modes when the network is driven by conflicting external input encoding multiple locations in the environment. In this situation, small megamaps operating in the WTA mode effectively suppress the input encoding one location and fully represent the second location, but large megamaps operating in the combinatorial mode robustly represent both locations through two co-stable activity bumps (Fig. 1(c) and (d)). Moreover, hysteresis is observed only in the WTA mode, and a megamap in the combinatorial mode linearly amplifies the difference in input strengths (Fig. 3(a) and (c)). In our simulations with $N\approx 10\mbox{,}000$ place cells, the transition between modes occurs when the learned region reaches about 25 m^2^ [10].

The combinatorial mode is not commonly observed in attractor network models. Standard continuous attractor network models of place cells operate exclusively in the WTA mode unless the dynamical system is modified to make multi-peaked activity bumps more stable [6, 37, 38]. It is interesting that the optimal megamap operates in either mode without any changes to the parameters or dynamical system, but the megamap with Hebbian weights operates in the WTA mode regardless of the environmental size. The emergence of the combinatorial mode not only depends on the environmental size but also on the manner in which the recurrent connections are updated as the animal explores novel regions of the environment.

### Numerical Test for the Operational Mode

We now propose a numerical test for determining the operational mode of the dynamical system (Eq. (1)). We specify that the system is in the combinatorial mode if there exist stable fixed points with multiple activity bumps, and the network is in the WTA mode if any stable fixed point has exactly one activity bump.

We find that the stability of any fixed point depends on the subset of active cells at the fixed point, or excitatory cells such that $f(u_{i})>0$ and the inhibitory unit (inh) when $f^{\mathrm {I}}(\textbf{u})>0$. We define *S* and $S^{\mathrm{I}}$ as the sets of active excitatory and inhibitory cells, respectively, and prove in Appendix A that the fixed point is stable if and only if $r(S,S^{\mathrm{I}})<1$, where

3 $$r\left(S,S^{I}\right)\equiv \lambda_{\max}\left(f_{\mathrm{pk}}\left(\text{W}-\chi_{S^{I}}(\mathrm{inh})w^{I}11^{T}\right)\text{D}(S)\right).$$

Here, $\lambda_{\max}(\textbf{M})$ refers to the largest real part of all eigenvalues of the matrix **M**, $\mbox{\raisebox{1pt}{$\chi$}}_{S^{\mathrm{I}}}(\mathrm{inh})$ is the indicator function for the set $S^{\mathrm{I}}$ (1 if the inhibitory unit is active and 0 otherwise), and $\textbf{D}(S)$ is the diagonal (0–1)-matrix with $\textbf{D}_{ii}(S) = \mbox{\raisebox{1pt}{$\chi$}}_{S}(i)$ (1 if $i\in S$ and 0 otherwise). Note that the stability depends only on the weights (**W** and $w^{\mathrm{I}}$) and on which cells are active. The external input and the magnitude of each state do not affect the stability of a fixed point.

To determine the operational mode, we randomly select two well-separated locations in the environment (at least 50 cm apart and at least 15 cm from an environmental boundary). Let $\textbf{x}_{1}$ and $\textbf{x}_{2}$ denote these two locations, and let $\overline {S_{k}}$ denote the set of all active cells in the embedded activity bump over $\textbf{x}_{k}$ (Eq. (2)), or

4 $$ \overline {S_{k}} = \bigl\{ i : \overline {f}_{i}(\textbf{x}_{k})>0\bigr\} $$

for $k=1,2$. Since $\theta<1$, the inhibitory unit is active given any embedded activity bump. In our numerical simulations, the inhibitory unit is always active at an equilibrium state regardless of the external input. Hence, we set $S^{\mathrm{I}}= \{\mathrm{inh}\} $. According to our test, the system is in the combinatorial mode if and only if $r(\overline {S_{1}}\cup \overline {S_{2}},{\{\mathrm{inh}\} })<1$. This test is accurate when there exists a fixed point with two bumps in which the set of active excitatory cells is the set of excitatory cells that are active in either embedded activity pattern, or $S= \overline {S_{1}}\cup \overline {S_{2}}$. The activity pattern at such a fixed point is approximated by a linear combination of the two embedded activity bumps, or $f(\textbf{u}) \approx c_{1}\overline {\textbf{f}}(\textbf{x}_{1})+c_{2}\overline {\textbf{f}}(\textbf{x}_{2})$ for some positive constants $c_{1}$ and $c_{2}$ such that $c_{1}+c_{2}>\theta$.

In all numerical simulations we performed, the test is accurate in distinguishing between the two operational modes. For the example presented in Fig. 1, the recurrent weight matrix **W** is updated as the animal gradually learns novel subregions of an environment [10]. For the optimal weights, the test predicts the transition from the WTA mode to the combinatorial mode as the area (*A*) of the learned environment grows. In particular, $r(\overline {S_{1}}\cup \overline {S_{2}},{\{\mathrm{inh}\} })$ decreases as *A* becomes larger, dropping below 1 around 25 m^2^ (Fig. 1(b), black closed circles). As predicted, when $A<25\mbox{ m}^{2}$, exactly one activity bump persists in time given any initial state and any external input (Fig. 1(c)). When $A>25\mbox{ m}^{2}$, two activity bumps persist in time given a mixed external input (Fig. 1(d)). For the Hebbian weights, the test predicts that the system remains in the WTA mode regardless of *A* since $r( \overline {S_{1}}\cup \overline {S_{2}},{\{\mathrm{inh}\} })$ gradually increases with *A* (Fig. 1(b), gray closed circles). As predicted, we find numerically that two activity bumps are always unstable given Hebbian weights [10].

Equation (3) can also be used to test the stability of single-peaked fixed points. Regardless of *A* or the method used to set the weights, $r(\overline {S_{k}},{\{\mathrm{inh}\} })<1$ for any location $\textbf {x}_{k}$ (Fig. 1(b), open circles and diamonds)). This indicates that any single-peaked fixed point proportional to an embedded activity bump is stable. It is important to note that even in the combinatorial mode, the system robustly represents any location through a stable single-peaked activity bump given a single-peaked external input that may be relatively weak, noisy, or incomplete.

The numerical test is a powerful tool for determining the behavior of the network *a priori*. In addition to determining whether it is possible for multiple activity bumps to persist in time, the test determines whether the network may show hysteresis or amplify the difference in input strengths (Fig. 3(a) and (c)). However, the numerical test is limited in that it determines the stability but not the existence of a fixed point. Figure 1(b), open circles and diamonds, indicates that single-peaked activity bumps are stable for any size environment. In our numerical simulations, we found that these single-peaked fixed points always exist given the optimal weights, but all cells eventually become active when $A=625\mbox{ m}^{2}$ given Hebbian weights [10]. Some sort of normalization, such as forcing the 1-norm (subtractive normalization) or 2-norm (multiplicative normalization) of the weight vector to be constant, would be required to maintain stability in the Hebbian network [33]. It would be interesting to examine in future work how normalization would affect the operational mode of the Hebbian network.

## 2-Unit Reduced Model

While the numerical test of Eq. (3) can be used to determine the operational mode, we seek a deeper understanding of why the operational mode emerges in large environments, and under what set of parameters. We begin by reducing the model to a simple 2-unit model that has similar dynamics and we can fully analyze.

### Reduction of the Megamap Model to the 2-Unit Model

Consider an external input that is some mixture of the two training inputs, $\overline {\textbf{b}}(\textbf{x}_{1})$ and $\overline {\textbf{b}}(\textbf{x}_{2})$ (Eq. (2)), where $\textbf{x}_{1}$ and $\textbf{x}_{2}$ are two well-separated locations in the environment. We seek a mapping from the full megamap model to a two-dimensional reduced model with the same form and the same qualitative dynamics given this conflicting external input. The simplest relevant simplification is to model two units, where the place cells in each unit *k* are given by the set $\overline {S_{k}}$ (Eq. (4)), and the reduced state $\widehat {u}_{k}$ is the collective state of place cells in unit *k*. The reduced model does not include cells without a place field near $\textbf{x}_{1}$ or $\textbf{x}_{2}$, as these cells should be silent ($f(u_{i})\approx0$) if the system is stable.

**Fig. 2:**
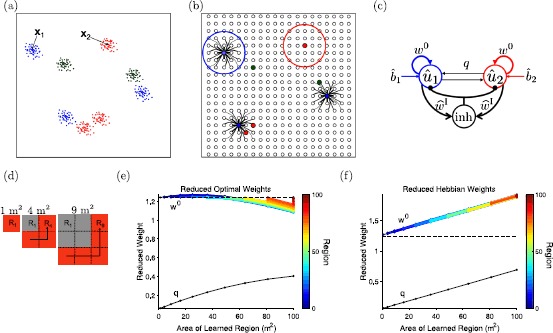
Reduction of the megamap model to the 2-unit model. (**a**) Schematic showing idealized place fields of three different place cells, where the green cell has two place fields, and the red and blue cells each have three place fields. In the megamap model, the place fields of each cell are set randomly according to the Poisson distribution. The two-unit model is an approximation of the megamap driven by an external input encoding two locations, denoted by $\textbf{x}_{1}$ and $\textbf{x}_{2}$. (**b**) Each place cell is plotted redundantly on the megamap at each of its preferred locations. For both the optimal and the Hebbian megamaps, each place cell has recurrent connections to each set of its neighbors. Idealized connections from the blue cell are shown. The place cells inside the large blue and red circles are the cells included in unit 1 and unit 2, respectively. (**c**) The two-unit model (Eq. (5)) has the same form as the megamap model (Eq. (1)). The reduced state variables and reduced external input, $\widehat {u}_{k}$ and $\widehat {b}_{k}$ (Eq. (6)), represent the collective state and collective external input into place cells near location $\textbf{x}_{k}$, indicated by the blue and red circles in (**b**). The reduced weights, $w^{0}$ and *q* (Eq. (7)), are related to the strength of connections within a unit and between units, respectively. For this example, there should be a relatively weak cross-connection *q* since the blue and red cells are neighbors elsewhere in the environment. The reduced inhibitory weight is proportional to the inhibitory weight of the megamap (Eq. (7)). (**d**)–(**f**) We compute the reduced weights for a megamap that models an animal incrementally learning a square environment of increasing size [10]. The first three iterations are illustrated in (**d**). At each iteration, the recurrent weights are updated to incorporate the novel subregions (red) into the learned environment (gray). Previously learned subregions are not reinforced in later iterations. For the optimal weights (**e**), the average recurrent excitation (proportional to $w^{0}$) within a unit changes little over the first 100 m^2^ compared to the increase in the average weight between units (proportional to *q*) as the environment grows in size. For the Hebbian weights (**f**), $w^{0}$ and *q* increase linearly at roughly the same rate. The color in (**e**) and (**f**) indicates the region number (the first nine regions are shown in (**d**))

The reduction is illustrated in Fig. 2(a)–(c). Explicit equations for the reduced 2-unit model are given by Eqs. (5)–(7). The weights of the reduced model, $w^{0}$ and *q*, are directly related to the weights of the full megamap model. For example, consider the three cells whose place fields within the environment are illustrated in Fig. 2(a) by the colors blue (Cell 1), red (Cell 2), and green (Cell 3). Each cell is plotted redundantly on the megamap at each of its preferred locations (Fig. 2(b)). If the external input innervates cells near locations $\textbf{x}_{1}$ and $\textbf{x}_{2}$ indicated in (a), then the cells enclosed by the blue and red circles in (b) are collectively represented by units 1 and 2, respectively. The reduced weight $w^{0}$ determines the degree to which cells within a unit reinforce each other’s activity and is related to the weights among cells in a unit on the megamap. The reduced weight *q* determines the degree to which cells within one unit innervate cells in a different unit and is proportional to the average weight between cells in different units on the megamap. If each cell had only a single place field, then there would be no cross-excitation, or $q=0$. Due to the multiplicity of place fields, however, two cells in different units may innervate each other due to overlapping place fields elsewhere in the environment. In the example shown, $q>0$ since Cells 1 and 2 are neighbors on the megamap. We thus expect $0< q< w^{0}$, since only some of the cells in the two units have overlapping place fields.

**Fig. 3:**
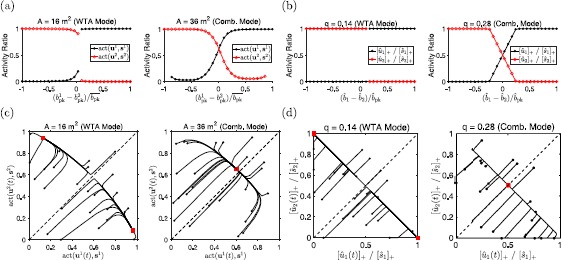
Comparison of the operational modes of the full and reduced models. (**a**) The full megamap with optimal weights is driven by the conflicting external input, $\textbf{b} = (1/\overline {b_{\mathrm{pk}}})(b_{\mathrm {pk}}^{1}\overline {\textbf{b}}(\textbf{x}_{1}) + b_{\mathrm{pk}}^{2}\overline {\textbf{b}}(\textbf{x}_{2}))$, where $\overline {\textbf {b}}(\textbf{x}_{k})$ is the training input into location $\textbf{x}_{k}$ (Eq. (2)). For the relatively small megamap operating in the WTA mode (left, Fig. 1(c)), any equilibrium activity bump fully represents one location while effectively ignoring the input for the other location. For the large megamap operating in the combinatorial mode (right, Fig. 1(d)), the equilibrium activity bump fully represents one location when $|b_{\mathrm{pk}}^{1}-b_{\mathrm{pk}}^{2}|$ is sufficiently large. Otherwise, the equilibrium state corresponds to a linear combination of the two embedded activity bumps, amplifying the difference in input strengths. The initial state for all simulations corresponds to $\overline {\textbf{f}}( \textbf{x}_{2})$ (Eq. (2)). The activity ratio is given by $\operatorname{act}(\textbf{u}^{k},\textbf{s}^{k}) \equiv\sum_{i\in \overline {S_{k}}}f(u_{i}) / \sum_{i\in \overline {S_{k}}} f(s^{k}_{i})$, where $u_{i}$ and $s_{i}^{k}$ are the equilibrium states of cell *i* given the conflicting external input, $b_{i}$, and the isolated input, $( b_{\mathrm{pk}}^{k}/\overline {b_{\mathrm{pk}}})\overline {b}_{i}(\textbf{x}_{k})$, respectively. Data points were omitted if $f(\textbf{s}^{k})$ was not an activity bump over location $\textbf{x}_{k}$, which occurs in this example when $b_{\mathrm{pk}}^{k}\approx0$. (**b**) The 2-unit model responds similarly to the conflicting external input. The parameters $w^{0}=1.2$ and *q* are comparable to the corresponding reduced weights of the megamap (Eq. (7), Fig. 2(e)). The reduced inhibitory weight, $\widehat {w}^{\mathrm{I}}= 5.3$, and threshold, $\theta=0.9$, are the exact values corresponding to the megamap parameters in (**a**). (**c**) The initial state (black circles) is varied randomly, and the external input is constant ($b_{\mathrm{pk}}^{1}=b_{\mathrm{pk}}^{2}=\overline {b_{\mathrm{pk}}}/2$). The equilibrium state reached (red squares) depends on the initial state for the small megamap but not for the large megamap. (**d**) The 2-unit model with the same parameters as used in (**b**) similarly shows hysteresis only in the WTA mode. Here, $\widehat {b}_{1}= \widehat {b}_{2}=\widehat {b}_{\mathrm{pk}}/2$

Figure 2(e) and (f) shows $w^{0}$ and *q* (Eq. (7)) for a megamap representing square environments of increasing size (Fig. 2(d)). This megamap was used to generate Figs. 1, 2, 3, and further details on its construction and behavior can be found in [10]. For the Hebbian megamap, new weights are added as the animal explores new locations. This results in a linear increase in both $w^{0}$ and *q* as the environment grows in size, but a constant difference, $w^{0}-q$ (Fig. 2(f)). For the optimal megamap, weights are both increased and decreased so that each novel subregion is accurately learned. As a result, $w^{0}$ is constant for the most recent 1 m^2^ subregion learned. While the reduced weight $w^{0}$ within a given subregion gradually decays as new subregions are incorporated into the megamap, $w^{0}$ changes little compared to the increase in *q* over the initial 100 m^2^ (Fig. 2(e)). The steady decrease in $w^{0}-q$ is correlated to the decrease in the dominant eigenvalue (Fig. 1(b), closed black circles) and appears to be responsible for the change in operational mode. We prove this is the case in Sect. 4.1.

#### Reduced Model

We now present explicit equations for the reduced model. As shown in Appendix B, computing the sum over all cells in unit *k* ($\overline {S_{k}}$) of each term in Eq. (1) and scaling by $(f_{\mathrm{pk}}/\overline {f_{\mathrm {net}}})$ leads to the two-dimensional reduced model,

5 $$ \left \{ \textstyle\begin{array}{l} \tau \widehat {u}_{1}'(t) = -\widehat {u}_{1}(t) + w^{0}[\widehat {u}_{1}(t)]_{+} + q[\widehat {u}_{2}(t)]_{+}\\ \quad{}-\widehat {w}^{\mathrm{I}} [[\widehat {u}_{1}(t)]_{+} + [\widehat {u}_{2}(t)]_{+} - \theta]_{+} + \widehat {b}_{1},\\ \tau \widehat {u}_{2}'(t) = -\widehat {u}_{2}(t) + q[\widehat {u}_{1}(t)]_{+} + w^{0}[\widehat {u}_{2}(t)]_{+} \\ \quad{}- \widehat {w}^{\mathrm{I}} [[\widehat {u}_{1}(t)]_{+} + [\widehat {u}_{2}(t)]_{+} - \theta]_{+} + \widehat {b}_{2}. \end{array}\displaystyle \right . $$

The reduced model has the same form as the full megamap model, but the network connections are now defined by only two weights ($w^{0}$ and *q*) rather than the weight matrix $\textbf {W}\in \mathbb{R}^{N\times N}$. For simplicity, the activation function of the megamap, $f(u_{i}) = f_{\mathrm{pk}}[u_{i}]_{+}$, is scaled in the reduced model to have a peak value of 1. The two reduced state variables and corresponding external inputs are given by

6 $$ \widehat {u}_{k}(t) \equiv\frac{f_{\mathrm{pk}}}{\overline {f_{\mathrm{net}}}} \sum _{i\in \overline {S_{k}}} u_{i}(t) \quad\mathrm{and}\quad \widehat {b}_{k} \equiv \frac{f_{\mathrm{pk}}}{\overline {f_{\mathrm{net}}}} \sum_{i\in \overline {S_{k}}} b_{i} $$

for each unit *k*. When there is an activity bump over $\textbf{x}_{k}$ with the same radius as the embedded activity bump, $f(u_{i})\approx u_{\mathrm {pk}}\overline {f}_{i}(\textbf{x}_{k})$ for $i\in \overline {S_{k}}$, where $0< u_{\mathrm{pk}}\leq 1$ is the peak of the state bump. In this case, $\widehat {u}_{k}\approx u_{\mathrm{pk}}$, and so the embedded activity bump over $\textbf{x}_{k}$ maps to the reduced activity, $[\widehat {u}_{k}]_{+} = \widehat {u}_{k} \approx u_{\mathrm{pk}}$. When there is no activity bump over $\textbf{x}_{k}$, unit *k* is silent ($[\widehat {u}_{k}]_{+}=0$) since $u_{i}<0$ for most cells in unit *k*. The external input is always nonnegative, and it is zero when there is no external input into place cells in unit *k*.

The reduced weights are given by

7 $$ \begin{aligned} w^{0} &= \frac{f_{\mathrm{pk}}}{\overline {f_{\mathrm{net}}}} \sum_{i\in \overline {S_{1}}} \sum _{j\in \overline {S_{1}}} w_{ij} \overline {f}_{j}( \textbf{x}_{1}),\qquad q = \frac{f_{\mathrm{pk}}}{\overline {N}} \sum_{i\in \overline {S_{1}}} \sum_{j\in \overline {S_{2}}} w_{ij},\quad\mathrm{and}\\ \widehat {w}^{\mathrm{I}}&= f_{\mathrm{pk}}\overline {N} w^{\mathrm{I}}, \end{aligned} $$

where *N̅* denotes the average number of active cells in each embedded activity pattern, so $\overline {N}\approx|\overline {S_{k}}|$ for any *k*. In our simulations of the megamap, $220\leq{|\overline {S_{k}}|}\leq225$ for all locations *k*. The weight of the self-connection ($w^{0}$) is proportional to the average recurrent excitation between two place cells in the same unit *k* given the embedded activity bump over $\textbf{x}_{k}$ (Eq. (2)), and the weight of the cross-connection (*q*) is proportional to the average weight between two place cells in different units. The reduced inhibitory weight is proportional to the inhibitory weight of the megamap. The inhibition into any reduced unit (*Î*) and the inhibition into any excitatory cell in the megamap ($I = w^{\mathrm{I}}f^{\mathrm{I}}(\textbf{u})$) are related by

$$\widehat {I} = \frac{f_{\mathrm{pk}}}{\overline {f_{\mathrm{net}}}}\sum_{i\in \overline {S_{1}}} I = \frac{f_{\mathrm{pk}}\overline {N}}{\overline {f_{\mathrm{net}}}}I. $$

Consequently, the inhibitory unit is active in the 2-unit model if and only if the inhibitory unit is active in the full megamap model, and the inhibition drives the state of an inactive unit further below zero for the 2-unit model than for the megamap model since $f_{\mathrm {pk}}\overline {N}>\overline {f_{\mathrm{net}}}$.

#### Approximations in the Reduction

As detailed in Appendix B, we make four approximations to map the *N*-dimensional system of Eq. (1) to the two-dimensional system of Eq. (5). First, we neglect cells that are in both units by assuming $\overline {S_{1}}\cap \overline {S_{2}}= \emptyset$. Since place fields are set by the Poisson distribution, a small minority of cells in $\overline {S_{1}}$ may also be in $\overline {S_{2}}$, but these relatively few cells should not have a large impact on the dynamics. Second, we neglect the small minority of cells with multiple place fields near $\textbf{x}_{k}$. This permits the assumptions that both units have the same number of cells, or $\overline {N}= |\overline {S_{k}}|$ for any *k*, and that the average of the recurrent input (proportional to $w^{0}$) between two cells in the same unit given the embedded activity bump is the same for all *k*. Third, we neglect the asymmetries in the optimal weights of the megamap by assuming that the average weight from unit 1 to unit 2 (proportional to *q*) is the same as the average weight from unit 2 to unit 1. These first three approximations amount to neglecting the variability of the megamap and modeling only the average dynamics. The variability may affect the stability of a state in borderline cases. For example, when $r( \overline {S_{1}}\cup \overline {S_{2}},{\{\mathrm{inh}\} }) \approx1$, the stability of two co-active bumps may depend on the locations chosen for $\textbf{x}_{1}$ and $\textbf{x}_{2}$.

The fourth approximation does affect the average dynamics of the megamap. We assume that any activity bump over $\textbf{x}_{k}$ has the same radius and is always centered over $\textbf{x}_{k}$. Explicitly, we define $S_{k}(t)$ as the set of all cells near $\textbf{x}_{k}$ that are active at time *t*, or

8 $$ S_{k}(t) \equiv\Bigl\{ i : \bigl(u_{i}(t)>0\bigr) \text{ and }\Bigl(\min_{m} |\textbf{x}_{k}- \textbf{c}_{im}|< \delta\Bigr) \Bigr\} . $$

(The exact value of *δ* is not important here. It should be larger than the radius of the embedded activity bump, and small enough to exclude cells that are active due to their proximity to the location of the other unit.) To obtain Eq. (5), we assume $S_{k}(t)\in\{\emptyset, \overline {S_{k}}\}$ for all *t*, where $S_{k}\approx \emptyset$ when there is no activity bump over $\textbf{x}_{k}$, and $S_{k}\approx \overline {S_{k}}$ when there is an activity bump over $\textbf{x}_{k}$. In reality, the radius expands continuously from 0 to its equilibrium value as an activity bump emerges. We are perhaps justified in neglecting these transient, narrow activity bumps since we use the 2-unit model to infer the stable fixed points of the megamap. However, in the absence of external input, the equilibrium activity bump drifts over the megamap [10], so it is important to choose $\textbf{x}_{k}$ to be a location from which activity bumps do not drift. In addition, the equilibrium activity bump is wider for weaker external inputs. The 2-unit model does not capture the effects of a wider activity bump, but rather tracks only the height of the activity bump since $S_{k}\approx \overline {S_{k}}\Rightarrow \widehat {u}_{k}\approx u_{\mathrm {pk}}$. Despite this shortcoming, we find that the two models behave in the same way qualitatively (Fig. 3), and the analytical tractability of the 2-unit model permits us to derive explicit equations for the set of parameters leading to each operational mode and the relative strength of external input leading to hysteresis (in the WTA mode) or two co-stable activity bumps (in the combinatorial mode).

### Constraints on the Parameters of the 2-Unit Model

In accordance with the construction of the megamap with optimal weights, the parameters of the 2-unit model are set such that when the network is driven by the training inputs, $[\widehat {b}_{\mathrm{pk}} \ 0] ^{\mathsf{T}}$ and $[0 \ \widehat {b}_{\mathrm{pk}}] ^{\mathsf{T}}$, the respective fixed points of Eq. (5) correspond to the desired activity patterns, $[1\ 0] ^{\mathsf{T}}$ and $[0\ 1] ^{\mathsf{T}}$, respectively. The training input strength, $\widehat {b}_{\mathrm {pk}}$, is proportional to the parameter $\overline {b_{\mathrm{pk}}}$ in the megamap model (Eq. (2) and Eq. (6)). These two desired activity patterns are obtained if and only if

9 $$ 1 = w^{0} - \widehat {w}^{\mathrm{I}}(1-\theta)+\widehat {b}_{\mathrm{pk}}. $$

We set $w^{0}$, $\widehat {w}^{\mathrm{I}}$, and *θ* as the parameters of the 2-unit model, and we analyze its behavior as we vary *q*, $\widehat {b}_{1}$, and $\widehat {b}_{2}$. All parameters and variables are nonnegative and must satisfy the following constraints:

1. The inhibitory unit must be active given a desired activity pattern, but inactive if all place cells are inactive. Equivalently, $0<\theta<1$.
2. The strength of the training input must be much weaker than the desired equilibrium state, or $0<\widehat {b}_{\mathrm{pk}}\ll1$. By Eq. (9), this condition is equivalent to $\widehat {w}^{\mathrm {I}}(1-\theta) \ll w^{0} < 1+\widehat {w}^{\mathrm{I}}(1-\theta)$.
3. When $q=0$, the attractor of the megamap should consist of single-peaked activity bumps. In the 2-unit model, this means that when $q=0$ and $\widehat {\textbf{b}}=\textbf{0}$, the system supports fixed points in which exactly one unit is active. Without loss of generality, suppose that the fixed point in the absence of external input is given by $\widehat {u}_{1}>0$ and $\widehat {u}_{2}<0$. We show in Appendix C.2 that the inhibitory unit must be active at such a fixed point. By Eq. (5),
  $$ \begin{bmatrix} \widehat {u}_{1}\\ \widehat {u}_{2} \end{bmatrix} = \begin{bmatrix}w^{0}&0\\ 0&w^{0} \end{bmatrix} \begin{bmatrix} \widehat {u}_{1}\\ 0 \end{bmatrix} - \widehat {w}^{\mathrm{I}}( \widehat {u}_{1}-\theta) \begin{bmatrix}1\\1 \end{bmatrix} \quad \Rightarrow\quad w^{0}-1 = -\widehat {u}_{2}/\widehat {u}_{1}. $$
  Thus, this condition imposes the constraint, $w^{0}>1$.
4. Finally, the cross-excitation must be small enough such that the desired activity pattern is a fixed point of the system given the training input. With $\widehat {b}_{1}=\widehat {b}_{\mathrm{pk}}$ and $\widehat {b}_{2} = 0$, the fixed point must satisfy $\widehat {u}_{1}=1$ and $\widehat {u}_{2} = q -\widehat {w}^{\mathrm {I}}(1-\theta)<0$. Thus, this condition imposes the constraint, $q<\widehat {w}^{\mathrm{I}}(1-\theta) \ll w^{0}$.

## Analysis of the Operational Modes of the 2-Unit Model

In accordance with the definitions of the operational modes of the megamap, we specify that the 2-unit model is in the combinatorial mode if there exist stable fixed points in which both units are active and in the WTA mode if any stable fixed point has exactly one active unit. We now analyze the 2-unit model to derive an explicit equation for the critical value of $w^{0}-q$ at which the system shifts from the WTA mode to the combinatorial mode. We also analyze how the system responds to conflicting inputs in each mode, dependent on the attractor network strength ($w^{0}-q$) and the relative strengths of the competing inputs ($\widehat {b}_{1}- \widehat {b}_{2}$).

### Characterization of the Operational Modes

Assume the 2-unit network is driven by an external input of the form $\widehat {b}_{1}\geq \widehat {b}_{2}\geq0$. We derive all fixed points and analyze their stability in Appendices C and D, respectively. The main results are summarized below:

- At least one unit must be active at any stable fixed point due to the constraint, $w^{0}>1$.
- A fixed point in which only unit 1 is active exists if and only if
  10 $$ q < \bigl(w^{0}-1\bigr) + \frac{( \widehat {b}_{1}- \widehat {b}_{2})(\widehat {w}^{\mathrm {I}}-(w^{0}-1))}{\widehat {w}^{\mathrm{I}}\theta+ \widehat {b}_{1}}. $$
  Since $w^{0}-1<\widehat {w}^{\mathrm{I}}$, this fixed point exists for all inputs such that $\widehat {b}_{1}\geq \widehat {b}_{2}$ if and only if $w^{0}-q>1$. If the fixed point exists, it is always stable and corresponds to the network encoding only the location with the stronger external input ($\textbf {x}_{1}$). The network effectively ignores the weaker input over location $\textbf{x}_{2}$.
- A fixed point in which only unit 2 is active exists if and only if
  11 $$ q < \bigl(w^{0}-1\bigr) - \frac{( \widehat {b}_{1}- \widehat {b}_{2})(\widehat {w}^{\mathrm {I}}-(w^{0}-1))}{\widehat {w}^{\mathrm{I}}\theta+ \widehat {b}_{2}}. $$
  This fixed point exists for some input such that $\widehat {b}_{1}\geq \widehat {b}_{2}$ if and only if $w^{0}-q>1$. If the fixed point exists, it is always stable and corresponds to the network encoding only the location with the weaker external input ($\textbf{x}_{2}$). The network effectively ignores the stronger input over location $\textbf{x}_{1}$.
- A fixed point in which both units are active is stable if and only if $w^{0}-q < 1$. When $w^{0}-q<1$, such a fixed point exists if and only if
  12 $$ q > \bigl(w^{0}-1\bigr) + \frac{( \widehat {b}_{1}- \widehat {b}_{2})(\widehat {w}^{\mathrm {I}}-(w^{0}-1))}{\widehat {w}^{\mathrm{I}}\theta+ \widehat {b}_{1}}, $$
  and the fixed point is unique.

Explicit equations for all fixed points are given in Appendix C.

Setting $\widehat {b}_{1}= \widehat {b}_{2}$ in (Eq. (12)), we conclude that the system is in the WTA mode when $w^{0}-q>1$ and in the combinatorial mode when $w^{0}-q<1$. This result is consistent with the hypothesis that the shift in operational mode observed in the megamap is due to the increase in cross-excitation between cells in the two respective activity bumps (Fig. 2(e)). Although the inhibitory weight and threshold ($w^{\mathrm{I}}$ and *θ*, respectively) were not varied in our simulations of the megamap, the analysis of the 2-unit reduced model implies that the operational mode depends only on the difference in self- and cross-excitation, $w^{0}-q$, and not on $w^{\mathrm{I}}$ or *θ*. This is somewhat surprising since the competition between two activity bumps, which underlies the WTA mode, is mediated by feedback inhibition.

In the WTA mode of the 2-unit model, any stable fixed point represents exactly one location. This corresponds to the single-peaked activity bumps always observed in equilibrium states of a relatively small megamap (Fig. 1(c), Fig. 3(a) and (c)). Since Eq. (10) is always satisfied, there are two stable fixed points for a given set of inputs ($\widehat {b}_{1}\geq \widehat {b}_{2}$) if and only if Eq. (11) is satisfied. In this case, the equilibrium state reached depends on the initial state, consistent with the hysteresis observed in the WTA mode of the megamap model (Fig. 3(c)).

In the combinatorial mode of the 2-unit model, the stable fixed point represents only the stronger input when Eq. (10) is satisfied and both inputs when Eq. (12) is satisfied. This is consistent with the combinatorial mode of the megamap model, for which the equilibrium state always has one activity bump given a sufficiently large difference in input strengths and two activity bumps given two similar inputs (Fig. 3(a)). Since $q>w^{0}-1$, Eq. (11) is never satisfied, and the system never shows hysteresis. When both units are active in the equilibrium state, the state vector amplifies the difference in inputs according to

13 $$ \widehat {u}_{1}-\widehat {u}_{2} = \frac{ \widehat {b}_{1}- \widehat {b}_{2}}{q-(w^{0}-1)}. $$

The absence of hysteresis and the amplification of the difference in input strengths are both characteristic of the combinatorial mode of the megamap, as seen in the examples in Fig. 3(a) and (c).

### Bifurcations of the Dynamical System

Our analysis of the 2-unit model reveals four types of qualitative dynamics observed in the model:

- Type I: The state vector converges to a unique equilibrium in which only unit 1 is active.
- Type II: The state vector converges to a unique equilibrium in which only unit 2 is active.
- Type III: The state vector converges to one of two possible equilibria, one in which only unit 1 is active and one in which only unit 2 is active.
- Type IV: The state vector converges to a unique equilibrium in which both units are active.

**Fig. 4:**
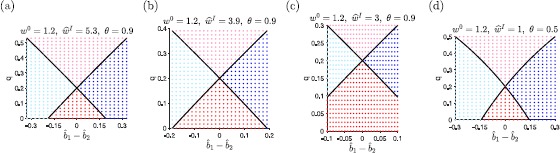
Bifurcations in the 2-unit model. Each plot shows the parameter sets $(\Delta \widehat {b}= \widehat {b}_{1}- \widehat {b}_{2}, q)$ leading to each type of qualitative dynamics described in Sect. 4.2. The 2-unit model shows hysteresis (Type III dynamics) when $q<(w^{0}-1)+g(-|\Delta \widehat {b}|)$, fully represents the location with the stronger input while suppressing the response to the weaker input (Type I or Type II dynamics) when $(w^{0}-1)+g(-|\Delta \widehat {b}|)< q<(w^{0}-1)+g(|\Delta \widehat {b}|)$, and linearly combines the two embedded activity patterns (Type IV dynamics) when $q>(w^{0}-1)+g(| \Delta \widehat {b}|)$. These bifurcations are shown by the solid black lines. The points specify the type of dynamics found in numerical simulations of the 2-unit model, where light blue (left region) indicates Type I, dark blue (right region) indicates Type II, dark red (bottom region) indicates Type III, and light red (top region) indicates Type IV. In all cases, the numerical simulations agree with the analytical predictions given by Eqs. (14)–(16). The initial state is set to the desired activity pattern such that the active unit is the unit driven by the weaker input. We classified the dynamics as Type I or Type II when the only active unit in the equilibrium state is the unit receiving the stronger input, as Type III when the initially active unit remains the only active unit in the equilibrium state, and as Type IV when both units are active in the equilibrium state. (**a**) The parameters of the 2-unit model approximate the reduced parameters from the megamap model (Eq. (7)), as used in Fig. 3(b) and (d). The four regions predict the response of the corresponding megamap as *q* and Δ*b̂* vary. (**b**) and (**c**) Bifurcations given a smaller reduced inhibitory weight. Reducing $\widehat {w}^{\mathrm{I}}$ reduces the range of permissible values for *q*, shrinking the relative size of the parameter space with Type IV dynamics compared to that with Type III dynamics. The transition between operational modes ($q=0.2$) is not affected by $\widehat {w}^{\mathrm{I}}$. (**d**) Bifurcations given a smaller inhibitory threshold, which makes the nonlinearity in $g(x)$ more apparent. The full ranges of permissible *q* and Δ*b̂* are shown for each plot

We have already shown that Types I, II, and III are found in the WTA mode, while Types I, II, and IV are found in the combinatorial mode. We now derive explicit equations for the bifurcations, or parameter sets on the boundary between two different types of qualitative dynamics, in order to better understand the interplay between the inherent strength of the attractor network ($w_{0}-q$) and the relative strength of external inputs ($\Delta \widehat {b}\equiv \widehat {b}_{1}- \widehat {b}_{2}$). To simplify analysis, we assume the net external input is constant, or $\widehat {b}_{1}+ \widehat {b}_{2}=\widehat {b}_{\mathrm {pk}}$. As the learned environment grows from 0 to about 100 m^2^, the only parameter in the optimal megamap with large relative changes is *q* (Fig. 2(e)). Hence, we hold the parameters $w^{0}, \widehat {w}^{\mathrm {I}}$, and *θ* fixed and determine the bifurcations for the parameters $0\leq q < \widehat {w}^{\mathrm{I}}(1-\theta)$ and $-\widehat {b}_{\mathrm{pk}} \leq \Delta \widehat {b}\leq \widehat {b}_{\mathrm{pk}}$, where $\widehat {b}_{\mathrm{pk}}$ is given by Eq. (9). Examples of bifurcations are shown in Fig. 4.

By substituting the constraint $\widehat {b}_{1}+ \widehat {b}_{2}=\widehat {b}_{\mathrm{pk}}= \widehat {w}^{\mathrm {I}}(1-\theta)-(w^{0}-1)$ and $\widehat {b}_{1}- \widehat {b}_{2}=\Delta \widehat {b}$ into the bounds in Eqs. (10)–(12), the bifurcations can all be expressed in terms of the function

14 $$ g(x)\equiv2x \biggl(\frac{\widehat {w}^{\mathrm{I}}-(w^{0}-1)}{\widehat {w}^{\mathrm{I}}(1+\theta)-(w^{0}-1)+x} \biggr) $$

over the domain $-\widehat {b}_{\mathrm{pk}}\leq x \leq \widehat {b}_{\mathrm {pk}}$. Since $w^{0}-1<\widehat {w}^{\mathrm{I}}$, *g* is strictly increasing over its domain, and $g(0)=0$.

By Eq. (11), the system has Type III dynamics (hysteresis) if and only if

15 $$ q < \bigl(w^{0}-1\bigr) + g\bigl(-|\Delta \widehat {b}|\bigr), $$

which is only possible in the WTA mode since $g(-|\Delta \widehat {b}|)\leq0$. As illustrated in Fig. 4, when the external input into one unit becomes sufficiently stronger than the other, then only the unit receiving the stronger input will remain active in the equilibrium state as the system transitions to Type I or Type II dynamics. As *q* becomes larger for a fixed $\Delta \widehat {b}\neq0$, the active unit increasingly depolarizes the silent unit. If this cross-excitation becomes sufficiently strong, it becomes impossible to maintain an activity bump over the unit receiving less input, again pushing the system into Type I or Type II dynamics.

By Eq. (12), the system has Type IV dynamics (two co-stable activity bumps) if and only if

16 $$ q > \bigl(w^{0}-1\bigr) + g\bigl(|\Delta \widehat {b}|\bigr), $$

which is only possible in the combinatorial mode since $g(|\Delta \widehat {b}|)\geq 0$. As illustrated in Fig. 4, the system again transitions to Type I or Type II dynamics when the external input into one unit becomes sufficiently stronger than the other. However, increasing *q* now causes a transition from uni-peaked equilibrium states of Type I or Type II to multi-peaked equilibrium states of Type IV. Increased cross-excitation between the units causes the units to better reinforce one another, counteracting the competition between units induced by feedback inhibition.

The bifurcations appear roughly linear for a wide range of weights $w^{0}$ and $\widehat {w}^{\mathrm{I}}$ when $\theta=0.9$ (Fig. 4(a)–(c)). To examine this, let $d(x)$ denote the denominator in Eq. (14). Since $-\widehat {b}_{\mathrm{pk}}\leq \Delta \widehat {b}\leq \widehat {b}_{\mathrm{pk}}$ and $\widehat {b}_{\mathrm{pk}}< \widehat {w}^{\mathrm{I}}(1-\theta)$,

$$2\widehat {w}^{\mathrm{I}}\theta- \bigl(w^{0}-1\bigr) \leq d\bigl(-|\Delta \widehat {b}|\bigr) \leq \widehat {w}^{\mathrm{I}}(1+\theta)-\bigl(w^{0}-1\bigr) \leq d\bigl(|\Delta \widehat {b}|\bigr) \leq2\widehat {w}^{\mathrm{I}}-\bigl(w^{0}-1\bigr). $$

Hence, $g(x)$ approaches a linear function with slope $(\widehat {w}^{\mathrm{I}}-(w^{0}-1) ) / (\widehat {w}^{\mathrm {I}}- (w^{0}-1)/2 )$ as *θ* approaches 1. The nonlinearities in $g(x)$ are more apparent for smaller values of *θ*. Figure 4(d) shows an example with $\theta=0.5$.

## Conclusions

We present a mathematical analysis of the properties of the megamap attractor neural network that emerge when the network represents a sufficiently large spatial environment [10]. Through stability analysis of the full megamap model, we derive a numerical test (Eq. (3)) for determining the operational mode of the dynamical system (Eq. (1)). In addition, we derive a linear mapping from the *N*-dimensional megamap model to a two-dimensional reduced model that has the same qualitative dynamics. Our analysis of the 2-unit model elucidates the role of each parameter in the full megamap model in the context of conflicting external inputs (Fig. 4). In particular, we show that the abrupt shift in operational mode occurs when $q \approx w^{0}-1$, where $w^{0}$ and *q* are proportional to the average recurrent excitation between two cells in the same unit and in different units, respectively (Eq. (7)). The inhibitory weight does not affect the operational mode, but increasing $w^{\mathrm{I}}$ increases the range of *q*, resulting in a larger range of the relative strength of inputs ($b_{\mathrm{pk}}^{1}-b_{\mathrm{pk}}^{2}$) for which there are two co-stable activity bumps (Type IV dynamics). The inhibitory threshold (*θ*) also does not affect the operational mode, but the bifurcations described by Eqs. (14)–(16) approach linear functions of $b_{\mathrm{pk}} ^{1}-b_{\mathrm{pk}}^{2}$ as *θ* approaches 1.

This work is similar in nature to numerous theoretical studies of EI nets [39, 40]. In many of these studies, two populations of neurons are considered, where one population represents excitatory cells and the other inhibitory cells. The recurrent circuitry among inhibitory cells is often neglected, simplifying the analysis. We consider two populations of excitatory neurons, each with extensive recurrent circuitry, and a third population of inhibitory neurons. We simplify the dynamical system by lumping all inhibitory neurons into a single inhibitory unit under the assumption that all inhibitory cells are statistically identical since interneurons in the hippocampus do not appear to have strong spatial tuning [41, 42]. We also assume that the time constant of the inhibitory state is much smaller than that of excitatory cells, allowing us to approximate the inhibitory state as an instantaneous function of the excitatory activity vector. Without this simplification, it is likely that we would observe oscillations between activity bumps under some parameter sets [18].

A common approach used to analyze continuous attractor neural networks is to approximate the *N*-dimensional system of ordinary differential equations (Eq. (1)) by a partial differential equation by taking the limit as $N\rightarrow\infty$. The state vector, $\textbf{u}(t)\in \mathbb{R}^{N}$, then becomes the continuous function, $u(\textbf{x},t)\in \mathbb{R}$, where **x** is a continuous variable representing the single preferred location of a given place cell. The cleanest results are obtained using a Heaviside activation function for $f(u)$, for then one can solve for the radius of the activity bump at a fixed point [14, 43]. Using a similar approach, we derived clean expressions for the set of stable fixed points; however, we found that the combinatorial mode does not exist given the Heaviside activation function in our dynamical system. Other mathematical studies have used Fourier analysis to analyze the PDE given the threshold linear activation function used for the megamap model [40, 44]. Even when we approximate the recurrent weights using only the first two terms in the Fourier series, however, the recurrent circuitry among both populations of neurons renders the solutions too complex to be helpful in understanding how the parameters of the model affect the dynamics. The approaches we present in this study require only a few justified approximations of the full megamap model, and the simplicity of the results make the analysis useful in understanding the behavior of the megamap. Despite its simplicity, the numerical test accurately determines the operational mode of the full system (Fig. 1), and the reduced model has similar qualitative behavior to the full model (Figs. 2 and 3).

While we focus on a particular attractor neural network, the results apply to a broad class of attractor network models. The numerical test for determining the operational mode (Eq. (3)) applies to any attractor network model in which the state vector is governed by Eq. (1), a standard firing rate model derived by averaging neuronal activity over multiple trials [33]. The reduced 2-unit model applies to any attractor neural network in which the four approximations outlined in Sect. 3.1 are valid approximations. This includes not only continuous attractor neural networks, but also discrete attractor neural networks such as Hopfield networks with graded neuronal responses [2]. In the latter case, the set $\overline {S_{k}}$ used in the reduction of the full model is the set of all cells that are active in embedded activity pattern *k*. It is not necessary for the embedded activity patterns to have the shape of the Gaussian-like activity bumps considered here.

When considering the reduced model, it is important to understand the impact of the approximations underlying the linear mapping from the full model. For the megamap, the first three approximations neglect the variability in embedded activity patterns and weights due to the Poisson distribution of place fields [10]. This variability includes asymmetries in the full weight matrix, **W**. We find numerically that, as long as **W** is a relatively small perturbation from a symmetric matrix, the asymmetries have a negligible effect on the dynamics. For example, we observe only a slight difference in the transition point between operational modes determined by numerical simulations and the stability test (∼25 m^2^) and by the reduced state variables ($w^{0}-q\approx1.05$ at 25 m^2^, as seen in Fig. 2(e)). This result is not surprising, as uncorrelated random perturbations of the weight matrix in a Hopfield network have been shown to have a small effect on the dynamics [45, 46]. The fourth approximation underlies the qualitative differences between the full megamap model and the 2-unit model. In particular, the variable radius of the activity bump underlies the nonlinearities observed in the megamap’s response to the conflicting input (Fig. 3(a) and (c)). In general, the reduced model captures the peak of the activity pattern, but it does not capture changes in the subset of active cells within each unit.

There are several natural directions in which the reduced model presented here could be extended. For example, one could examine how the attractor network responds to *M* conflicting external inputs, where $M\geq2$. As long as these inputs are well-separated spatially, an *M*-dimensional reduced model could be derived exactly as shown for $M=2$ in Sect. 3.1. Using the same four approximations, the reduced model for *M* inputs would be

$$\begin{aligned} \tau \widehat {u}_{k}'(t) ={}& {-}\widehat {u}_{k}(t) + \bigl(w^{0}-q\bigr) \bigl[\widehat {u}_{k}(t) \bigr]_{+} + q\sum _{j=1}^{M} \bigl[\widehat {u}_{j}(t) \bigr]_{+} \\&- \widehat {w}^{\mathrm{I}} \Biggl[\sum_{j=1}^{M} \bigl[u_{j}(t) \bigr]_{+} - \theta\Biggr]_{+} + \widehat {b}_{k} \quad \text{for }1\leq k\leq M. \end{aligned} $$

The reduction equations (Eqs. (6)–(7)) and the four constraints would be unchanged. There are several intriguing questions that could be addressed by this model. For example, does the value of *q* at which there exists a fixed point with $m\geq 2$ stable, co-active units depend on *m*? If so, the definition of the combinatorial mode would need to be reconsidered. Another interesting question is whether hysteresis emerges in the combinatorial mode when $M>2$. For example, it is possible that, for a particular parameter set, any stable fixed point has two co-active units, but the subset of co-active units depends on the initial state.

A second possible extension would be to relax the fourth approximation of the reduced model to examine the spatial effects of the activity bump on the attractor. This could be done by modeling $n\ll N$ place cells for each unit, setting the reduced weight matrix $\textbf {W}^{0}\in \mathbb{R}^{n\times n}$ through a Gaussian tuning curve, and setting the reduced weight matrix $\textbf{Q}\in \mathbb{R}^{n\times n}$ as a random matrix with $\|\textbf{Q}\|\ll\|\textbf{W}^{0}\|$. It would be interesting to compare the operational modes and bifurcations of this 2*n*-dimensional model to the operational modes and bifurcations of the two-dimensional model presented here.

A third possible extension would be to use the reduced model to explore remapping. In the current study, the full weight matrix is set during a learning phase in which the place cell activity is fixed at the desired activity pattern, and the network is driven by strong external inputs. Then the dynamics of the model are examined during a retrieval phase in which the weights are constant, and the recurrent input is stronger than the external input. This separation into a learning phase and retrieval phase is common in attractor neural network models in which the weights are incrementally learned [6, 35, 47], and there is experimental evidence supporting, at least in part, the use of two separate phases. For example, it has been observed experimentally that the acetylcholine system is more activated during the initial exploration of a novel space than when the animal is moving around in a familiar space, and acetylcholine may increase the strength of afferent input connections relative to feedback recurrent connections [48]. Nonetheless, it would be an interesting and relevant study to address how the dynamics change given plasticity in the recurrent weights during the retrieval phase, as is more biologically realistic. Exploring remapping mathematically would require a more complex reduced model that incorporates differential equations for $w^{0}(t)$ and $q(t)$. The basic Hebbian learning rule is unstable, and the manner in which stability is maintained would affect the set of stable fixed points [33]. Another key factor would be the learning rate. In particular, when the two external inputs have equal strength, then two activity bumps initially become co-active in the WTA mode when the weights are constant. In the full model, this co-activity could last for hundreds of ms before one activity bump dominates [10]. Given Hebbian learning, the place cells in each unit would begin to reinforce each other’s activity, effectively increasing *q* and possibly driving the system to the combinatorial mode.

There are many contexts in which an attractor neural network must resolve conflicting information from its rich array of neuronal inputs. For example, it is a common experimental paradigm to manipulate different cues in different ways in order to track how information flows through various levels of neural processing [49, 50]. The WTA mode is ideal for robust memory retrieval, allowing the attractor network to perform computations such as transforming a noisy external input into a coherent, embedded activity pattern. On the other hand, the combinatorial mode permits a flexible recombination of embedded activity patterns in response to a changed environment. This flexibility could lead to phenomena such as the partial remapping observed in hippocampal place cells [6, 10, 31]. Perhaps the ideal attractor neural network operates between these two extremes, robustly encoding memories while still having the flexibility to adapt to our ever-changing world. The reduction method presented in this paper is a useful tool for simplifying the mathematical analysis of various behaviors of attractor network models to better understand how these behaviors depend on the network parameters and the learning process.

## Appendix A: Stability of a Fixed Point of the Megamap Model

Let **v** be a fixed point of the dynamical system (Eq. (1)). Suppose that the state is perturbed from this fixed point at time $t_{0}$, so that $\textbf{u}(t) = \textbf{v}+ \epsilon \widetilde {\textbf{v}}(t)$ for some small $\epsilon>0$. Equation (1) becomes

17 $$\epsilon \tau {\tilde{\text{v}}}^{\prime }(t)=-\text{v}-\epsilon \tilde{\text{v}}(t)+\text{W}f\left(\text{v}+\epsilon \tilde{\text{v}}(t)\right)-w^{I}f^{I}\left(\text{v}+\epsilon \tilde{\text{v}}(t)\right)1+\text{b}.$$

The Taylor expansion of the activity of each place cell *i* is given by

$$f(v_{i}+\epsilon \widetilde {v}_{i}) = \left \{ \textstyle\begin{array}{l@{\quad}l} f(v_{i})&\mbox{if $v_{i}< 0$},\\ f(v_{i}) + \epsilon \widetilde {v_{i}}f_{\mathrm{pk}}+ O(\epsilon^{2}) & \mbox{if $v_{i}>0$}. \end{array}\displaystyle \right . $$

For simplicity, we assume that no state $v_{i}$ is exactly 0 and $\sum _{i=1}^{N} f(v_{i})$ is not exactly equal to $\theta \overline {f_{\mathrm {net}}}$. Let *S* denote the set of all active place cells at the fixed point, or the set of all excitatory cells *i* such that $v_{i}>0$. Define the diagonal (0–1)-matrix $\textbf{D}(S)\in \mathbb{R}^{N\times N}$ such that $D_{ii}(S) = \mbox{\raisebox{1pt}{$\chi$}} _{S}(i)$, where $\mbox{\raisebox{1pt}{$\chi$}}_{S}(i)$ is the indicator function, which takes a value of 1 when $i\in S$ and 0 otherwise. The recurrent input becomes

$$\textbf{W}f\bigl(\textbf{v}+\epsilon \widetilde {\textbf{v}}(t)\bigr) = \textbf{W}f( \textbf{v}) + \epsilon f_{\mathrm{pk}}\textbf{W}\textbf{D}(S)\widetilde {\textbf {v}}(t) + O\bigl( \epsilon^{2}\bigr). $$

The Taylor expansion of $f^{\mathrm{I}}$ about **v** is similarly given by

$$f^{\mathrm{I}}(\textbf{v}+\epsilon \widetilde {\textbf{v}}) = f^{\mathrm{I}}( \textbf{v}) + \epsilon \widetilde {\textbf{v}} \cdot \nabla f^{\mathrm {I}}(\textbf{v}) + O \bigl(\epsilon^{2}\bigr). $$

Let $S^{\mathrm{I}}$ denote the set of all active inhibitory cells at a fixed point. Since we model only one inhibitory unit (inh) representing the collective state of all inhibitory cells, $S^{\mathrm {I}}=\{\mathrm{inh}\}$ if $\sum_{i=1}^{N} f( v_{i})>\theta \overline {f_{\mathrm {net}}}$, and $S^{\mathrm{I}}=\emptyset$ otherwise. If $S^{\mathrm{I}}=\emptyset $, then $f^{\mathrm{I}}(\textbf{v}) = 0$ and $\nabla f^{\mathrm{I}}(\textbf{v}) = \textbf{0}$. Otherwise,

$$f^{I}(\text{v})=1^{T}f(\text{v})-\theta \bar{f_{\mathrm{net}}}\;\Rightarrow \;\frac{\partial f^{I}(\text{v})}{\partial v_{i}}=f^{\prime }(v_{i})=\chi_{S}(i)f_{\mathrm{pk}}.$$

Thus, $\tilde{\text{v}}\cdot \nabla f^{I}(\text{v})=\chi_{S^{I}}(\mathrm{inh})f_{\mathrm{pk}}1^{T}\text{D}(S)\tilde{\text{v}}$. Substituting these expressions back into Eq. (17) and dropping terms of $O(\epsilon^{2})$, we find

$$\begin{array}{rl} \tau {\tilde{\text{v}}}^{\prime }(t) & =-\tilde{\text{v}}(t)+f_{\mathrm{pk}}\text{W}\text{D}(S)\tilde{\text{v}}(t)-\chi_{S^{I}}(\mathrm{inh})f_{\mathrm{pk}}w^{I}11^{T}\text{D}(S)\tilde{\text{v}}(t),\\ & ⇓\\ \tau {\tilde{\text{v}}}^{\prime }(t) & =\left(-\text{I}+f_{\mathrm{pk}}\left(\text{W}-\chi_{S^{I}}(\mathrm{inh})w^{I}11^{T}\right)\text{D}(S)\right)\tilde{\text{v}}(t). \end{array}$$

Therefore, $\widetilde {\textbf{v}}(t)\rightarrow\textbf{0}$ if and only if

$$\lambda_{\max}\left(-\text{I}+f_{\mathrm{pk}}\left(\text{W}-\chi_{S^{I}}(\mathrm{inh})w^{I}11^{T}\right)\text{D}(S)\right)<0,$$

where $\lambda_{\max}(\textbf{M})$ specifies the largest real part of all eigenvalues of **M**. In conclusion, any fixed point with active excitatory and inhibitory cells *S* and $S^{\mathrm{I}}$, respectively, is stable if and only if $r(S,S^{\mathrm{I}})<1$, where

$$r\left(S,S^{I}\right)=\lambda_{\max}\left(f_{\mathrm{pk}}\left(\text{W}-\chi_{S^{I}}(\mathrm{inh})w^{I}11^{T}\right)\text{D}(S)\right).$$

This equation provides a numerical test to determine the stability of any fixed point.

## Appendix B: Reduction of the Megamap

In this appendix, we map Eq. (1) to Eq. (5) using the approximations described in Sect. 3.1. By Eq. (1),

$$\begin{aligned} \frac{f_{\mathrm{pk}}}{\overline {f_{\mathrm{net}}}} \sum _{i\in \overline {S_{1}}} \bigl(\tau u_{i}'(t) \bigr) &= \frac{f_{\mathrm{pk}}}{\overline {f_{\mathrm{net}}}} \sum_{i\in \overline {S_{1}}} \Biggl(-u_{i}(t) + \sum_{j=1}^{N} w_{ij} f \bigl(u_{j}(t) \bigr)\\&\quad- w^{\mathrm{I}} \Biggl[\sum _{j=1}^{N} f\bigl(u_{j}(t)\bigr) -\theta \overline {f_{\mathrm{net}}}\Biggr]_{+} + b_{i} \Biggr) \\ &\Downarrow \\ \tau \widehat {u}_{1}'(t) &= -\widehat {u}_{1}(t) + \frac{f_{\mathrm{pk}}}{\overline {f_{\mathrm{net}}}} \sum_{i\in \overline {S_{1}}} \sum _{j=1}^{N} w_{ij}f\bigl(u_{j}(t) \bigr) \\&\quad- \frac{f_{\mathrm{pk}}\overline {N}}{\overline {f_{\mathrm{net}}} }w^{\mathrm{I}} \Biggl[\sum _{j=1}^{N} f\bigl(u_{j}(t)\bigr)-\theta \overline {f_{\mathrm{net}}}\Biggr]_{+} + \widehat {b}_{1}, \end{aligned} $$

by Eq. (6), where $\overline {N}\approx| \overline {S_{1}}|\approx|\overline {S_{2}}|$. Let $S_{k}(t)$ be the set of all active cells near $\textbf{x}_{k}$ at time *t* (Eq. (8)). Assuming $S_{1}(t)\cap S_{2}(t)\approx\emptyset $, the inhibition into unit 1 becomes

$$\begin{aligned} &\frac{\widehat {w}^{\mathrm{I}}}{\overline {f_{\mathrm{net}}}} \Biggl[\sum _{j=1}^{N} f \bigl(u_{j}(t)\bigr)-\theta \overline {f_{\mathrm{net}}}\Biggr]_{+} \\&\quad \approx \widehat {w}^{\mathrm{I}} \biggl[\frac{1}{\overline {f_{\mathrm{net}}}} \sum _{j\in S_{1}(t)} f_{\mathrm{pk}}u_{j}(t) + \frac{1}{\overline {f_{\mathrm{net}}}} \sum _{j\in S_{2}(t)}f_{\mathrm{pk}}u_{j}(t) - \theta\biggr]_{+}, \end{aligned} $$

where $\widehat {w}^{\mathrm{I}}$ is given by Eq. (7). For each unit *k*, if there is no activity bump over $\textbf{x}_{k}$ at time *t*, then $S_{k}{(t)}\approx\emptyset$ and $\widehat {u}_{k}{(t)<} 0$, implying that

$$\frac{1}{\overline {f_{\mathrm{net}}}} \sum_{j\in S_{k}{(t)}} f_{\mathrm{pk}} u_{j}{(t)} \approx0 = \bigl[\widehat {u}_{k}{(t)}\bigr]_{+}. $$

If there is an activity bump over $\textbf{x}_{k}$ at time *t*, then we assume $S_{k}{(t)}\approx \overline {S_{k}}$ and $\widehat {u}_{k}(t)>0$, implying that

$$\frac{1}{\overline {f_{\mathrm{net}}}} \sum_{j\in S_{k}{(t)}} f_{\mathrm{pk}} u_{j}{(t)} \approx\frac{f_{\mathrm{pk}}}{\overline {f_{\mathrm{net}}}} \sum _{j\in \overline {S_{k}}} u_{j}{(t)} = \widehat {u}_{k}{(t)} = \bigl[ \widehat {u}_{k}{(t)}\bigr]_{+}. $$

Hence, the inhibition into unit 1 is approximated by $\widehat {w}^{\mathrm {I}} [[\widehat {u}_{1}(t)]_{+} + [\widehat {u}_{2}(t)]_{+} - \theta]_{+}$, the inhibition in Eq. (5).

All that remains is to show that the network input into unit 1 is approximated by $w^{0}[\widehat {u}_{1}]_{+} + q[\widehat {u}_{2}]_{+}$. Again assuming $S_{1}(t)\cap S_{2}(t)\approx\emptyset$, the network input becomes

$$\frac{f_{\mathrm{pk}}}{\overline {f_{\mathrm{net}}}} \sum_{i\in \overline {S_{1}}}\sum _{j=1}^{N} w_{ij}f\bigl(u_{j}(t) \bigr) \approx R_{1}(t)+R_{2}(t), $$

where

$$R_{k}(t)\equiv\frac{f_{\mathrm{pk}}}{\overline {f_{\mathrm{net}}}} \sum _{i\in \overline {S_{1}}} \sum_{j\in S_{k}(t)} w_{ij}f\bigl(u_{j}(t) \bigr) \quad\mbox{for $k=1,2$.} $$

If there is no activity bump over $\textbf{x}_{1}$ at time *t*, then $S_{1}{(t)}\approx\emptyset$ and $\widehat {u}_{1}{(t)}<0$, implying that $R_{1}{(t)} \approx0 = w^{0}[\widehat {u}_{1}{(t)}]_{+}$. Similarly, if there is no activity bump over $\textbf{x}_{2}$ at time *t*, then $R_{2}{(t)}\approx0 = q[\widehat {u}_{2}{(t)}]_{+}$. If there is an activity bump over $\textbf{x}_{1}$ at time *t*, we assume $S_{1}{(t)}\approx \overline {S_{1}}$. This implies that $f(u_{j}{(t)}) \approx u_{\mathrm{pk}}{(t)} \overline {f}_{j}(\textbf{x}_{1})$ and $[\widehat {u}_{1} ]_{+} = \widehat {u} _{1}{(t)} \approx u_{\mathrm{pk}}{(t)}$, where $u_{\mathrm{pk}}$ is the peak of the state bump over $\textbf{x}_{1}$. In this case,

$$R_{1}{(t)} \approx\frac{f_{\mathrm{pk}}}{\overline {f_{\mathrm{net}}}} \sum _{i\in \overline {S_{1}}}\sum_{j\in \overline {S_{1}}} w_{ij} \bigl(u_{\mathrm{pk}}{(t)} \overline {f}_{j}(\textbf{x}_{1}) \bigr) {= w^{0} u_{\mathrm{pk}}(t)} \approx w^{0} \bigl[\widehat {u}_{1}{(t)} \bigr]_{+} $$

by Eq. (7). Similarly, if there is an activity bump over $\textbf{x}_{2}$ at time *t*, then we assume $S_{2}{(t)}\approx \overline {S_{2}}$ and $\widehat {u} _{2}(t)>0$, implying that

$$R_{2}{(t)} \approx\frac{f_{\mathrm{pk}}}{\overline {f_{\mathrm{net}}}} \biggl(\sum _{i\in \overline {S_{1}}}\sum_{j\in \overline {S_{2}}} w_{ij} \overline {f}_{j}(\textbf{x}_{2}) \biggr) {\bigl[}\widehat {u}_{2}{(t)} { \bigr]_{+}}. $$

It is reasonable to consider $w_{ij}$ and $\overline {f}_{j}(\textbf{x}_{2})$ as independent random variables for the following reason. The values of $w_{ij}$ and $\overline {f}_{j}$ depend on the preferred locations of cells *i* and *j*, which are Poisson random variables. Since $\overline {S_{1}}\cap \overline {S_{2}}\approx\emptyset$, when $i\in \overline {S_{1}}$ and $j\in \overline {S_{2}}$, $w_{ij}$ depends only on the place fields of cells *i* and *j* that are away from both $\textbf{x}_{1}$ and $\textbf{x}_{2}$, and $\overline {f}_{j}(\textbf{x}_{2})$ depends only on the place fields of cell *j* near $\textbf{x}_{2}$. Since all preferred locations are set as independent random variables, $w_{ij}$ and $\overline {f}_{j}(\textbf{x}_{2})$ are independent. Thus, $\text{E}[w_{ij}\overline {f}_{j}(\textbf{x}_{2})] = \text{E}[w_{ij}]\text{E}[\overline {f}_{j}(\textbf{x}_{2})]$, and so

$$\begin{aligned} R_{2}{(t)} &\approx\frac{f_{\mathrm{pk}}\overline {N}^{2}}{\overline {f_{\mathrm {net}}}} \biggl(\frac{1}{\overline {N}^{2}} \sum _{i\in \overline {S_{1}}}\sum_{j\in \overline {S_{2}}} w_{ij} \biggr) \biggl(\frac{1}{\overline {N}} \sum _{j\in \overline {S_{2}}} \overline {f}_{j}(\textbf{x}_{2}) \biggr) {\bigl[} \widehat {u}_{2}{(t)\bigr]_{+}}\\& = \frac{f_{\mathrm{pk}}\overline {N}^{2}}{\overline {f_{\mathrm {net}}}} \biggl(\frac{1}{\overline {N}^{2}} \sum _{i\in \overline {S_{1}}}\sum_{j\in \overline {S_{2}}} w_{ij} \biggr) \biggl(\frac{\overline {f_{\mathrm{net}}} }{\overline {N}} \biggr) {\bigl[} \widehat {u}_{2}{(t)\bigr]_{+}} = q\bigl[\widehat {u}_{2}{(t)}\bigr]_{+} \end{aligned} $$

by Eq. (7).

Putting it all together, we have derived a linear mapping from Eq. (1) to the differential equation,

$$\tau \widehat {u}_{1}'(t) \approx-\widehat {u}_{1}(t) + w^{0}\bigl[\widehat {u}_{1}(t)\bigr]_{+} + q\bigl[\widehat {u}_{2}(t) \bigr]_{+} - \widehat {w}^{\mathrm{I}} \bigl[\bigl[\widehat {u}_{1}(t)\bigr]_{+}+\bigl[ \widehat {u}_{2}(t)\bigr]_{+}-\theta\bigr]_{+} + \widehat {b}_{1}. $$

An analogous argument can be used to derive the equation governing $\widehat {u} _{2}$ (Eq. (5)).

## Appendix C: Fixed Points of the 2-Unit Model

A fixed point of the 2-unit model is any solution of the equation,

18 $$ \begin{bmatrix} \widehat {u}_{1}\\ \widehat {u}_{2} \end{bmatrix} = \begin{bmatrix}w^{0}&q\\ q&w^{0} \end{bmatrix} \begin{bmatrix} [\widehat {u}_{1} ]_{+}\\ [\widehat {u}_{2} ]_{+} \end{bmatrix} - \widehat {w}^{\mathrm{I}} \widehat {f}^{\mathrm{I}}(\widehat {\textbf{u}}) \begin{bmatrix}1\\1 \end{bmatrix} + \begin{bmatrix} \widehat {b}_{1}\\ \widehat {b}_{2} \end{bmatrix} , $$

where $\widehat {f}^{\mathrm{I}}(\widehat {\textbf{u}}) \equiv[ [\widehat {u} _{1}]_{+} + [\widehat {u}_{2}]_{+} - \theta]_{+}$. Without loss of generality, assume $\widehat {b}_{1}\geq \widehat {b}_{2}\geq0$. We also assume that all parameters satisfy the constraints outlined in Sect. 3.2.

### C.1 No Active Units

Suppose $\widehat {u}_{1}\leq0$ and $\widehat {u}_{2}\leq0$. Since $[\widehat {u}_{1}]_{+}=[\widehat {u} _{2}]_{+}=\widehat {f}^{\mathrm{I}}(\widehat {\textbf{u}}) = 0$, the only fixed point with no active unit is $\widehat {u}_{1}=\widehat {u}_{2}=0$, which exists if and only if $\widehat {b}_{1}= \widehat {b}_{2}=0$.

### C.2 One Active Unit

A fixed point with exactly one active unit corresponds to a single activity bump on the megamap. We will consider two cases separately.

#### Case 1

The unit receiving more input is the active unit, or $\widehat {u}_{1}>0$ and $\widehat {u}_{2} < 0$. By the second row of Eq. (18),

$$\widehat {u}_{2} = q\widehat {u}_{1} - \widehat {w}^{\mathrm{I}} \widehat {f}^{\mathrm{I}}(\widehat {\textbf{u}}) + \widehat {b}_{2}< 0\quad\Rightarrow \quad \widehat {f}^{\mathrm{I}}(\widehat {\textbf{u}}) > (q\widehat {u}_{1}+ \widehat {b}_{2})/\widehat {w}^{\mathrm{I}}\geq0. $$

Thus, $\widehat {f}^{\mathrm{I}}(\widehat {\textbf{u}}) = \widehat {u}_{1}-\theta>0$. Substituting this back into Eq. (18), we find

19 $$ \widehat {u}_{1} = \frac{\widehat {w}^{\mathrm{I}}\theta+ \widehat {b}_{1}}{\widehat {w}^{\mathrm {I}}-(w^{0}-1)} \quad\mathrm{and}\quad \widehat {u}_{2} = \bigl(q-\bigl(w^{0}-1\bigr) \bigr)\widehat {u}_{1}-( \widehat {b}_{1}- \widehat {b}_{2}). $$

Since $\widehat {u}_{1}>0$ for any permissible parameters, this fixed point exists if and only if $\widehat {u}_{2}<0$, or equivalently,

$$q < \bigl(w^{0}-1\bigr) + \frac{( \widehat {b}_{1}- \widehat {b}_{2})(\widehat {w}^{\mathrm {I}}-(w^{0}-1))}{\widehat {w}^{\mathrm{I}}\theta+ \widehat {b}_{1}}. $$

#### Case 2

The unit receiving less input is the active unit, or $\widehat {u}_{1}< 0$ and $\widehat {u}_{2}>0$. By the first row of Eq. (18),

$$\widehat {u}_{1} = q\widehat {u}_{2} - \widehat {w}^{\mathrm{I}} \widehat {f}^{\mathrm{I}}(\widehat {\textbf{u}}) + \widehat {b}_{1} < 0\quad\Rightarrow \quad \widehat {f}^{\mathrm{I}}(\widehat {\textbf{u}}) > (q\widehat {u}_{2}+ \widehat {b}_{1})/\widehat {w}^{\mathrm{I}}\geq0. $$

Thus, $\widehat {f}^{\mathrm{I}}(\widehat {\textbf{u}})=\widehat {u}_{2}-\theta>0$. Substituting this back into Eq. (18), we find

20 $$ \widehat {u}_{2} = \frac{\widehat {w}^{\mathrm{I}}\theta+ \widehat {b}_{2}}{\widehat {w}^{\mathrm {I}}-(w^{0}-1)}\quad\mathrm{and}\quad \widehat {u}_{1} = \bigl(q-\bigl(w^{0}-1\bigr) \bigr)\widehat {u}_{2} + ( \widehat {b}_{1}- \widehat {b}_{2}). $$

Since $\widehat {u}_{2}>0$ for any permissible parameters, this fixed point exists if and only if $\widehat {u}_{1}<0$, or equivalently,

$$q < \bigl(w^{0}-1\bigr) - \frac{( \widehat {b}_{1}- \widehat {b}_{2})(\widehat {w}^{\mathrm {I}}-(w^{0}-1))}{\widehat {w}^{\mathrm{I}}\theta+ \widehat {b}_{2}}. $$

### C.3 Two Active Units

A fixed point with two active units, or $\widehat {u}_{1}>0$ and $\widehat {u}_{2}>0$, corresponds to two activity bumps on the megamap, each encoding a different location in the environment.

Suppose the inhibitory unit is silent, or $\widehat {f}^{\mathrm{I}}(\widehat {\textbf{u}}) = 0$. Equation (18) becomes

$$ \begin{bmatrix}w^{0}-1 & q\\ q&w^{0}-1 \end{bmatrix} \begin{bmatrix} \widehat {u}_{1}\\\widehat {u}_{2} \end{bmatrix} = \begin{bmatrix}- \widehat {b}_{1}\\- \widehat {b}_{2} \end{bmatrix} . $$

If $q=w^{0}-1>0$, then

$$q \begin{bmatrix} \widehat {u}_{1}+\widehat {u}_{2}\\ \widehat {u}_{1}+\widehat {u}_{2} \end{bmatrix} = \begin{bmatrix}- \widehat {b}_{1}\\ - \widehat {b}_{2} \end{bmatrix} \quad\Rightarrow\quad \widehat {b}_{1}= \widehat {b}_{2}, \quad \mathrm{and}\quad \widehat {u}_{1}+\widehat {u}_{2} = - \widehat {b}_{1}/q{\leq}0. $$

The latter statement contradicts the assumption that $\widehat {u}_{1}>0$ and $\widehat {u} _{2}>0$. Thus, if $\widehat {f}^{\mathrm{I}}(\widehat {\textbf{u}})=0$, then $q\neq w^{0}-1$. In this case, the system has the unique fixed point,

$$\widehat {\textbf{u}} = \frac{1}{(w^{0}-1)^{2} - q^{2}} \begin{bmatrix}q \widehat {b}_{2}-(w^{0}-1) \widehat {b}_{1}\\ q \widehat {b}_{1}-(w^{0}-1) \widehat {b}_{2} \end{bmatrix} . $$

If $q< w^{0}-1$, then $\widehat {u}_{1} <0$ since $q \widehat {b}_{2}-(w^{0}-1) \widehat {b}_{1} < (w^{0}-1)(\widehat {b}_{2}- \widehat {b}_{1})\leq0$. If $q>w^{0}-1$, then $\widehat {u}_{2}<0$ since $q\widehat {b}_{1}-(w^{0}-1)\widehat {b}_{2} > (w^{0}-1)( \widehat {b}_{1}- \widehat {b}_{2})\geq0$. Since $\widehat {u}_{1}>0$ and $\widehat {u}_{2}>0$, the inhibitory unit must be active, or $\widehat {f}^{\mathrm{I}}(\widehat {\textbf{u}}) = \widehat {u}_{1}+\widehat {u}_{2}-\theta>0$.

Substituting this back into Eq. (18), the fixed point must satisfy

$$ \begin{bmatrix} \widehat {w}^{\mathrm{I}}-(w^{0}-1)&\widehat {w}^{\mathrm {I}}-q\\ \widehat {w}^{\mathrm{I}}-q&\widehat {w}^{\mathrm{I}}-(w^{0}-1) \end{bmatrix} \begin{bmatrix} \widehat {u}_{1}\\\widehat {u}_{2} \end{bmatrix} = \begin{bmatrix} \widehat {b}_{1}+\widehat {w}^{\mathrm{I}}\theta\\ \widehat {b}_{2}+\widehat {w}^{\mathrm{I}}\theta \end{bmatrix} . $$

The determinant of the coefficient matrix is given by $d = (q-(w^{0}-1))(2\widehat {w}^{\mathrm{I}}- (w^{0}-1)-q)$. Note that $2\widehat {w}^{\mathrm{I}}-(w^{0}-1)-q>0$.

If $q=w^{0}-1$, then a fixed point exists if and only if $\widehat {b}_{1}= \widehat {b}_{2}$. Under these conditions, the set of all fixed points is given by the line segment,

$$\widehat {u}_{1}+\widehat {u}_{2} ={\frac{ \widehat {b}_{1}+\widehat {w}^{\mathrm{I}}\theta }{\widehat {w}^{\mathrm{I}}-q}},\quad\text{where } \widehat {u}_{1}>0 \quad\mathrm{and}\quad \widehat {u}_{2}>0. $$

The inhibitory unit is active at the fixed point since ${\widehat {u}_{1}+\widehat {u}_{2}\geq\theta/(1-(q/\widehat {w}^{\mathrm{I}})) > \theta}$ given any set of permissible parameters.

If $q\neq w^{0}-1$, then the system has the unique fixed point,

21 $$ \widehat {\textbf{u}} = (1/d) \begin{bmatrix} \widehat {w}^{\mathrm{I}}\theta(q-(w^{0}-1))+ \widehat {b}_{1}(\widehat {w}^{\mathrm {I}}-(w^{0}-1))- \widehat {b}_{2}(\widehat {w}^{\mathrm{I}}-q)\\ \widehat {w}^{\mathrm{I}}\theta(q-(w^{0}-1))- \widehat {b}_{1}(\widehat {w}^{\mathrm {I}}-q)+ \widehat {b}_{2}(\widehat {w}^{\mathrm{I}}-(w^{0}-1)) \end{bmatrix} . $$

The inhibitory unit is again active at the fixed point given any permissible parameters since

$$\widehat {u}_{1}+\widehat {u}_{2} = \frac{2\widehat {w}^{\mathrm{I}}\theta+ \widehat {b}_{1}+\widehat {b}_{2}}{2\widehat {w}^{\mathrm{I}}-(w^{0}-1)-q} \geq\frac{\theta}{1-\frac {(w^{0}-1)+q}{2\widehat {w}^{\mathrm{I}}}} > \theta. $$

All that remains is to determine parameters for which $\widehat {u}_{1}>0$ and $\widehat {u} _{2}>0$. Consider the activity difference,

$$\widehat {u}_{1}-\widehat {u}_{2} = \frac{ \widehat {b}_{1}- \widehat {b}_{2}}{q-(w^{0}-1)}. $$

If $q< w^{0}-1$, then $\widehat {u}_{1}<\widehat {u}_{2}$ when $\widehat {b}_{1}> \widehat {b}_{2}$, implying that the unit receiving *less* input has a *higher* activity level at the unique fixed point. While such a fixed point may exist for certain parameters, we will later show that it is not stable. If $q>w^{0}-1$, then $\widehat {u}_{1}\geq \widehat {u}_{2}$, and by Eq. (21),

$$\begin{aligned} \widehat {u}_{2}>0 &\quad\Leftrightarrow\quad \widehat {w}^{\mathrm{I}}\theta \bigl(q-\bigl(w^{0}-1\bigr)\bigr)- \widehat {b}_{1}\bigl( \widehat {w}^{\mathrm{I}}-q\bigr)+ \widehat {b}_{2}\bigl(\widehat {w}^{\mathrm {I}}- \bigl(w^{0}-1\bigr)\bigr)>0 \\ &\quad\Leftrightarrow\quad q\bigl(\widehat {w}^{\mathrm{I}}\theta+ \widehat {b}_{1} \bigr)-\bigl(w^{0}-1\bigr) \bigl(\widehat {w}^{\mathrm{I}}\theta+ \widehat {b}_{2}\bigr)-\widehat {w}^{\mathrm {I}}( \widehat {b}_{1}- \widehat {b}_{2})>0 \\ &\quad\Leftrightarrow\quad q\bigl(\widehat {w}^{\mathrm{I}}\theta+ \widehat {b}_{1} \bigr) - \bigl(w^{0}-1\bigr) \bigl(\widehat {w}^{\mathrm{I}}\theta+ \widehat {b}_{1}\bigr)\\ &\phantom{\quad\Leftrightarrow\quad}\quad+\bigl(w^{0}-1\bigr) ( \widehat {b}_{1}- \widehat {b}_{2}) - \widehat {w}^{\mathrm{I}}( \widehat {b}_{1}- \widehat {b}_{2}) > 0 \\ &\quad\Leftrightarrow\quad q>\bigl(w^{0}-1\bigr)+ \biggl( \frac{\widehat {w}^{\mathrm{I}}-(w^{0}-1)}{\widehat {w}^{\mathrm{I}}\theta+ \widehat {b}_{1}} \biggr) ( \widehat {b}_{1}- \widehat {b}_{2}). \end{aligned}$$

Thus, this fixed point exists only for a sufficiently small input difference, $\widehat {b}_{1}- \widehat {b}_{2}$, or a sufficiently large weight between units, *q*.

## Appendix D: Stability of a Fixed Point of the 2-Unit Model

Since the 2-unit model has the same form as the megamap model, the stability of a fixed point in which $\widehat {u}_{1}\neq0$, $\widehat {u}_{2}\neq 0$, and $\widehat {u}_{1}+\widehat {u}_{2}\neq\theta$ is also determined by the stability test of Eq. (3), where *S* is now the set containing the indices of all active units, and $f_{\mathrm{pk}}=1$ due to the rescaled activation function in the reduced model. We now evaluate $r(S,S^{\mathrm{I}})$ to determine the stability of the various fixed points found in Appendix C (Eqs. (19)–(21)).

### D.4 No Active Units

The only fixed point with no active unit is $\widehat {u}_{1}=\widehat {u}_{2}=0$, which exists if and only if $\widehat {b}_{1}= \widehat {b}_{2}=0$. Since Eq. (3) does not apply for this fixed point, suppose the state is perturbed from the 0-vector at time $t_{0}$ such that $\widehat {u}_{1}(t_{0})>0$, $\widehat {u} _{2}(t_{0})>0$, and $\widehat {u}_{1}(t_{0})+\widehat {u}_{2}(t_{0})<\theta$. While both states are positive, the state vector is governed by

$$\tau \widehat {\textbf{u}}'(t) = -\widehat {\textbf{u}}(t) + \widehat {\textbf{W}} \widehat {\textbf{u}}(t) = (\widehat {\textbf{W}}-\textbf{I})\widehat {\textbf{u}}(t), $$

where **I** denotes the $2\times2$ identity matrix, and

$$ \widehat {\textbf{W}} \equiv \begin{bmatrix}w^{0}&q\\ q&w^{0} \end{bmatrix} . $$

The eigenvalues of $\widehat {\textbf{W}}-\textbf{I}$ are $\{(w^{0}-1)-q, (w^{0}-1)+q\}$. Since $w^{0}>1$, $w^{0}-1+q>0$, so the 0-state is not stable.

### D.5 One Active Unit

Without loss of generality, assume unit 1 is the active unit, or $\widehat {u} _{1}>0$ and $\widehat {u}_{2}<0$. At a fixed point, $\widehat {u}_{1}>\theta$, and so by Eq. (3), $\widehat {\textbf{u}}$ is stable if and only if $\lambda_{\max}(\textbf{M})<1$, where

$$\text{M}=\left(\hat{\text{W}}-{\hat{w}}^{I}11^{T}\right)\text{D}(S)=\left[\begin{array}{cc} w^{0}-{\hat{w}}^{I} & 0\\ q-{\hat{w}}^{I} & 0 \end{array}\right].$$

Since $w^{0}-\widehat {w}^{\mathrm{I}}< 1+\widehat {w}^{\mathrm{I}}(1-\theta) - \widehat {w}^{\mathrm{I}}< 1$, a fixed point with exactly one active unit is always stable.

### D.6 Two Active Units

Finally, suppose $\widehat {u}_{1}>0$ and $\widehat {u}_{2}>0$. Since $\widehat {f}^{\mathrm {I}}(\widehat {\textbf{u}})>0$ at the fixed point, the stability test is again given by $\lambda_{\max}(\textbf{M})<1$, where

$$\text{M}=\left(\hat{\text{W}}-{\hat{w}}^{I}11^{T}\right)\text{D}(S)=\left[\begin{array}{cc} w^{0}-{\hat{w}}^{I} & q-{\hat{w}}^{I}\\ q-{\hat{w}}^{I} & w^{0}-{\hat{w}}^{I} \end{array}\right].$$

For any set of permissible parameters, the fixed point is stable to even perturbations (in the direction of the eigenvector $\textbf{v}_{+} = [1\ 1] ^{\mathsf{T}}$) since $\lambda_{+} = w^{0}+q-2\widehat {w}^{\mathrm{I}}< 1$. However, the fixed point is stable to odd perturbations (in the direction of the eigenvector $\textbf{v}_{-} = [1 \ {-}1] ^{\mathsf{T}}$) if and only if $q>w^{0}-1$, since $\lambda_{-} = w^{0}-q$. Thus, for a fixed self-excitatory weight $w^{0}$, the system may transition from a mode in which this fixed point is unstable (WTA mode) to stable (combinatorial mode) as the cross-excitatory weight *q* increases.

## Abbreviations

WTA: winner-take-all mode. The WTA mode refers to an operational mode of the dynamical system in which any stable fixed point corresponds to exactly one activity bump.
Comb.: combinatorial. The comb. mode refers to an operational mode of the dynamical system in which there exist stable fixed points corresponding to multiple activity bumps.
